# Increased work of breathing and its relationship to dyspnea in malignant pleural effusion

**DOI:** 10.3389/fphys.2025.1664237

**Published:** 2025-12-19

**Authors:** Tomasz Gólczewski, Anna M. Stecka, Elżbieta M. Grabczak, Monika Zielińska-Krawczyk, Rafał Krenke

**Affiliations:** 1 Nalecz Institute of Biocybernetics and Biomedical Engineering, Polish Academy of Sciences, Warsaw, Poland; 2 Department of Internal Medicine, Pulmonary Diseases and Allergy, Medical University of Warsaw, Warsaw, Poland

**Keywords:** dyspnea, pleural effusion, pleural manometry, thoracentesis, work of breathing

## Abstract

**Objectives:**

Although dyspnea is the most common symptom of pleural effusion (PE), its physiological basis has not yet been fully elucidated. The aim of this work is to investigate the cause of dyspnea before therapeutic thoracentesis (TT) by analyzing the lack of dyspnea relief after TT.

**Methods:**

We retrospectively analyzed data gathered during TT. Among others, our database includes measurements of instantaneous pleural pressure (Ppl) in the ipsilateral hemithorax and airflow in the mouth (during TT), as well as arterial gas tensions (AGT) and dyspnea characterized quantitatively via the Modified Borg Scale (before and after TT). As the Borg scale is a subjective measure, the change in dyspnea (dB) was used in reliable quantitative analyses. Differences in various parameters and their changes between patients who reported dyspnea relief and the other patients (the YES and NO groups, respectively) were studied. Additionally, correlations between dB and these parameters (and their changes) were studied.

**Results:**

Only the amplitude of Ppl changes related to breathing after TT was significantly different (higher) in group NO than in group YES (p < 0.003; the large effect size). dB correlated with this amplitude and the volume of withdrawn fluid (r = −0.51 and 0.51, respectively), but it correlated with neither changes in AGT nor minute ventilation.

**Conclusion:**

The results suggest that the key mechanism of dyspnea in patients with malignant PE is related to reduced total lung compliance due to collapse of a lung part, leading to an increase in the work of breathing required to maintain adequate minute ventilation.

## 1 Introduction

Dyspnea is reported by approximately 50%–65% of patients with malignant pleural effusion (PE), compromising their quality of life and often being the primary reason for seeking emergency care (Antony et al., 2001; Piggott et al., 2023). Although various physiological mechanisms of dyspnea in patients with PE have been considered for decades, the key mechanism seems to remain unclear (Parshall et al., 2012; Thomas et al., 2015).

The accumulation of pleural fluid is associated with compression of the ipsilateral lung, which may result in increased ventilation‒perfusion mismatch and impaired gas exchange. However, neither low arterial O_2_ (PaO_2_) nor elevated CO_2_ (PaCO_2_) partial pressures can be responsible for dyspnea in patients with PE, as both may either decrease or increase after therapeutic thoracentesis (TT) despite dyspnea relief, and the changes in PaO_2_ and PaCO_2_ do not correlate (Karetzky et al., 1978; Brandstetter and Cohen, 1979; Agustí et al., 1997; Stecka et al., 2018; Muruganandan et al., 2020; Taylor et al., 2021; Zielinska-Krawczyk et al., 2022). Therefore, another cause of dyspnea in PE patients should be considered.

The hydrostatic pressure of the pleural fluid exerted on the ipsilateral hemidiaphragm may lead to flattening or even reversal, significantly impairing diaphragm function; likewise, a large volume of pleural fluid puts pressure on the chest wall (overstretching intercostal muscles) and mediastinum, resulting in its contralateral shift and changes in respiratory mechanics (Thomas et al., 2015; Skaarup et al., 2020). Recent studies have posited that dyspnea in PE may stem primarily from abnormal ipsilateral hemidiaphragm function. Consequently, the reduction in dyspnea following TT may be related to the restoration of the ipsilateral hemidiaphragm shape and function (Muruganandan et al., 2020; Skaarup et al., 2020; Muruganandan et al., 2023). This, however, has not been supported by a more recent study (Fjaellegaard et al., 2024). On the other hand, although dyspnea relief can be statistically correlated with improvement in the ipsilateral hemidiaphragm shape and function, these changes may not be causally linked, i.e., TT could influence both dyspnea and hemidiaphragm function independently. This could explain why dyspnea does not resolve in some patients despite the hemidiaphragm returning to its normal upward curve after TT (Muruganandan et al., 2020; Psallidas et al., 2017).

Given the difficulties in determining the physiological causes of dyspnea before TT, we propose a kind of reversal of the problem, i.e., investigation of the reasons for the lack of dyspnea relief after TT. We attempted to use our own database to study this problem. This database contains various data gathered during TT, including records of the pleural pressure (P_pl_) enabling the determination of the amplitude of P_pl_ changes related to breathing (P_pl_ampl_).

**FIGURE 1:**
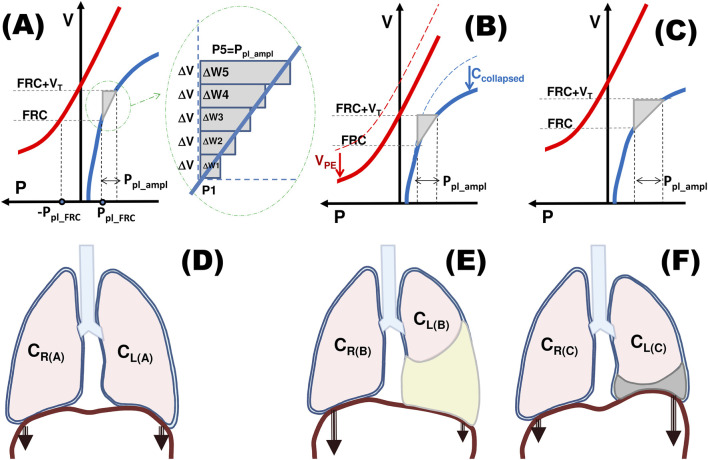
Influence of pleural effusion on work of breathing – schematic diagrams. Bold red and blue curves – schematic nonlinear pressure-volume curves for the chest wall and lungs, respectively, characterizing their static, nonlinear compliances; V – the current volume of the chest cavity/lungs; FRC – functional residual capacity; V_T_ – a fixed tidal volume; P_pl_ – pleural pressure, P_pl_FRC_ – P_pl_ at FRC; P – P_atm_-P_pl_ in the case of the chest wall and P_pl_-P_atm_ in the case of lungs, P_pl_ampl_ – the change in P_pl_ required to inspire V_T_; W – work done during one breath; V_PE_ – the pleural fluid volume; C_R_ and C_L_ – volume-dependent compliance of the right and left lung, respectively; C_collapsed_ – decrease in the (left) lung compliance caused by ‘elimination’ of a lung part due to its collapse. **(A)** To increase the lung volume by ΔV, respiratory muscles have to develop the pressure P1 that decreases P_pl_ to the value that corresponds to FRC+ΔV. According to the laws of physics, the work ΔW1 done by these muscles is equal to P1⋅ΔV. Further increase of V by next ΔV requires pressure P2 and the work ΔW2 is equal to P2⋅ΔV, etc. The total work done by the muscles to inspire V_T_ is the sum of W1, W2, W3 … Wn, i.e., it is equal to the area of the grey triangle that is equal to 0.5⋅V_T_⋅P_pl_ampl_. **(B)** Pleural fluid takes up space in the chest decreasing the chest cavity volume, which leads to a downward shift of the chest wall characteristics. Collapse of a lung part leads to compression of the lung characteristics. Although the above leads to a decrease in FRC, its value corresponds to smaller differential lung compliance. As a result, P_pl_ampl_ necessary for the required V_T_ and WOB (grey triangle area) are greater than in the healthy respiratory system. **(C)** Pleural fluid withdrawal causes return of the chest wall characteristics to the original position; however, if the collapsed lung part is not recruited, the characteristics of the lungs does not change. FRC increases to the value for which this characteristics is much more horizontal, i.e., the differential compliance is smaller. As a result, P_pl_ampl_ necessary for the required V_T_ and W are greater than before the fluid withdrawal. **(D–F)** present schematically contributions of individual lungs to the total lung compliance presented in **(A)**, **(B)**, **(C)**, respectively. The magnitude of arrows indicates contributions of individual hemidiaphragms to W. **(D)** The pressure-volume curve for lungs illustrates the sum of volume-dependent C_R_ and C_L_. **(E)** C_L(B)_ is smaller than C_L(A)_ by C_collapsed_ due to collapse of a lung part; therefore, W must increase to maintain V_T_. **(F)** If the atelectatic part of the left lung is not recruited, the ventilated part must overexpand to take place of withdrawn pleural fluid; therefore, since C_L_ is volume-dependent, C_L(C)_ is smaller than C_L(B)_. As a consequence, W is greater, not smaller, than before therapeutic thoracentesis.

As it is shown in Figure 1A, the work done against elastic forces during one breath is approximately equal to 0.5⋅P_pl_ampl_⋅V_T_. As the minute work, commonly called work of breathing (WOB), is equal to the work done in one breath multiplied by the respiratory rate (RR), the elastic WOB (WOB_e_) is equal to 0.5⋅P_pl_ampl_⋅V_T_⋅RR. Since the minute ventilation is equal to V_T_⋅RR, we have:

$${\text{WOB}}_{e}=0.5\cdot P_{\text{pl}\_\text{ampl}}\cdot \left(V_{T}\cdot \text{RR}\right)=0.5\cdot P_{\text{pl}\_\text{ampl}}\cdot V_{E}$$

Thus, if V_E_ remains relatively stable, P_pl_ampl_ can be used to characterize WOB_e_. Consequently, this study is particularly focused on analyzing the relationship between dyspnea and WOB_e_.

## 2 Materials and methods

### 2.1 Patients

This study involved a retrospective analysis of data obtained from a comprehensive prospective project in which multiple physiological parameters were measured before, during, and after TT. This project received approval from the Institutional Review Board (KB 105/2012) and was registered on ClinicalTrials.gov (NCT02192138). Medical procedures were conducted at the Department of Internal Medicine, Pulmonary Diseases and Allergy of the Medical University of Warsaw.

The data used for analysis were obtained from patients with malignant PE referred to the hospital for TT. The inclusion criteria were as follows: (1) age between 18 and 85 years, (2) symptomatic pleural effusion occupying at least one-third of the hemithorax determined by posteroanterior chest radiogram, (3) the severity of symptoms (including dyspnea) warranting TT, (4) absence of contraindication for TT, and (5) signed consent to participate in the study. The following exclusion criteria were applied: (1) poor general health condition warranting non-extension of the procedure, (2) mechanical ventilation due to respiratory failure, and (3) unstable hemodynamic or respiratory status not related to pleural effusion.

### 2.2 Measurements and parameters

TT was performed with the patient in a sitting position. The pleural fluid was withdrawn intermittently, with 1-min breaks for measurement purposes.

Dyspnea was assessed just before and after TT via the Modified 10-point Borg Scale. Dyspnea relief associated with TT was quantified by the difference (ΔB) between the Borg Scores before (B_pre_) and after TT (B_post_), i.e., ΔB = B_pre_− B_post_. The instantaneous values of P_pl_ in the ipsilateral hemithorax (digital pleural manometer, IBBE, Poland) and airflow through the mouth (modified LungTest 1,000 spirometer, MES, Poland) were recorded and synchronized for further analyses. V_E_ was calculated as the sum of tidal volumes during the period of the spirometric measurement divided by the duration of this period. The median value of P_pl_ampl_ after TT (P_pl_ampl post_) was construed as an index of WOB_e_ at the end of the procedure.

Arterial blood gases were measured 1 hour before and 1 hour after TT. Arterial blood samples were collected from the radial or ulnar artery of patients breathing ambient air. Blood samples were analyzed within 15 minutes after collection using the Blood Gas Analyzer ABL 800 FLEX (Medical ApS, Brønshøj, Denmark).

More details about patients and methods can be found in previous articles (e.g., Zielinska-Krawczyk et al., 2018; Zielinska-Krawczyk et al., 2022).

### 2.3 Data analyses

Since dyspnea is a subjective sensation and may be perceived and reported differently by each patient, the absolute value of dyspnea score might not be directly comparable between patients. The relative dyspnea index that are ΔB and ΔB/B_pre_ seem to be better parameters for objective quantitative analysis; therefore, they were compared with other parameters and their changes.

Since dyspnea relief after TT was observed in only some patients, we divided our patients into two groups: the YES group consisted of patients who reported dyspnea relief (i.e., ΔB>0) and the NO group consisted of the other patients (ΔB≤0). Then, we analyzed which of the following parameters (or their changes) differentiated these groups: PaO_2_, PaCO_2_, V_E_, the volume of withdrawn pleural fluid (V_w_) and P_pl_ampl post_.

If a patient reported insignificant dyspnea before TT, i.e., if B_pre_≤2, he/she could not report significant ΔB regardless of the true degree of dyspnea relief. This might distort possible correlations between ΔB and other parameters or their changes; therefore, the main analyses were performed for patients who reported B_pre_>2, however correlations were also calculated for all patients.

### 2.4 Statistical methods

Statistical analyses were performed via the Statistica 10 package (StatSoft Inc.). As some of the analyzed data had distributions different from the normal distribution, nonparametric statistical methods were used. Statistical significance was considered when p < 0.05. The Spearman correlation coefficient was used to estimate associations between the analyzed parameters. The difference in parameters between the NO and YES groups was assessed via the Mann–Whitney U test, and the Glass rank-biserial correlation (rg) was used to estimate the effect size; if rg > 0.45, then the effect size was interpreted as large.

## 3 Results

**FIGURE 2:**
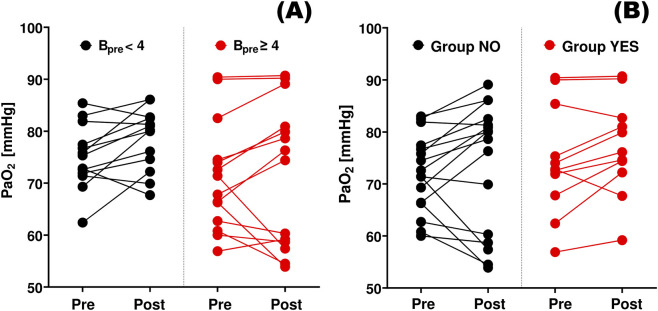
Changes in arterial oxygen tension. PaO_2 pre_ and PaO_2 post_ – the arterial oxygen tensions measured before (Pre) and after (Post) thoracentesis, respectively. **(A)** B_pre_ < 4: patients who reported no to moderate dyspnea before thoracentesis (Borg score <4); B_pre_ ≥ 4: patients with more severe dyspnea. **(B)** Group NO: patients who reported no dyspnea relief or worsening after therapeutic thoracentesis, Group YES – patients who reported dyspnea relief.

In general, data for 41 patients were analyzed; however, due to technical issues, arterial gasometry could not be done before or after TT in 14 patients. Table 1 presents the characteristics of the whole sample, and the YES and NO groups separately. Neither arterial blood gases (Figure 2B) nor V_E_ nor their changes differed among these groups (Table 1). B _pre_ and V_w_ were greater in the YES group; nevertheless, these differences were statistically insignificant. Moreover, although dispersion of PaO_2_ values was smaller in patients who reported not severe dyspnea before TT, neither the median values nor PaO_2_ changes were different (Figure 2A).

**TABLE 1:** Characteristics of the groups.

Parameter	All patients	Group NO (ΔB≤0)	Group YES (ΔB>0)	p
N	41	15	26	
Age [yrs]	66 (57; 77)	64 (42; 78)	66 (58; 77)	0.38
V_w_ [L]	1.8 (1.25; 2.3)	1.35 (1.0; 1.95)	1.9 (1.3; 2.7)	0.08
PaO_2 pre_ [mmHg]	72.6 (66.3; 75.6)	72.6 (63.8; 75.3)	72,0 (66.3; 75.8)	0.91
PaO_2 post_ [mmHg]	76.3 (65.6; 81.3)	78.0 (74.4; 82.7)	76.3 (59.7; 80.9)	0.25
ΔPaO_2_ [mmHg]	3.1 (−1.5; 6.6)	3.1 (0.2; 5.9)	3.1 (−1.5; 6.6)	0.86
PaCO_2 pre_ [mmHg]	35.8 (34.0; 38.8)	36.0 (34.0; 39.0)	35.7 (34.0; 38.1)	0.68
PaCO_2post_ [mmHg]	37.2 (35.0; 39.0)	36.2 (35.1; 37.7)	37.5 (34.3; 39.1)	0.44
ΔPaCO_2_ [mmHg]	0.55 (−1.1; 1.9)	−0.15 (−1.6; 1.3)	0.65 (−0.6; 2.5)	0.49
B _pre_	4 (3; 5)	3 (2; 4)	4.5 (3; 7)	0.07
B _post_	3 (1; 4)	4 (3; 5)	2 (1; 3)	- -
ΔB	1 (0; 3)	0 (−1; 0)	2.5 (1; 4)	- -
P_pl_ampl_ _post_ [cmH_2_O]<	12.3 (9.5;21.1)	19.2 (11.7;39.1)	11.3 (8.6;14.7)	0.003 (rg > 0.47)
V_E pre_ [l/min]	10.4 (9.4; 11.4)	10.0 (9.4; 11.4)	10.6 (9.3; 11.4)	0.76
V_E post_ [l/min]	10.0 (8.6; 11.6)	10.2 (8.4; 12.6)	9.9 (8.6; 11.3)	0.72
ΔV_E_% [%]	−1.2 (−12.3; 5.9)	1.2 (−10.3; 11.4)	−3.3 (−14.3; 4.5)	0.37

Group YES – patients who reported dyspnea relief after TT; Group NO – the other patients. N – number of patients; subscripts “pre” and “post” – the value of a parameter before (or at the beginning of) and after (or at the end of) TT, respectively; V_w_ – the volume of withdrawn fluid; PaO_2_, PaCO_2_ – arterial tensions of O_2_ and CO_2_, respectively; B – dyspnea quantified with the Borg scale; P_pl_ampl_ – the amplitude of pleural pressure changes related to breathing; V_E_ – minute ventilation. Δ, the change in the parameter value: ΔB – dyspnea relief (B_pre_-B_post_), for the other parameters: the “post” value minus the “pre” value. Data are presented as medians, and the 1^st^ and 3^rd^ quartiles in parentheses. P – the statistical significance of differences between groups NO, and YES (the Mann‒Whitney U test); rg – the effect size (the Glass rank-biserial correlation).

**FIGURE 3:**
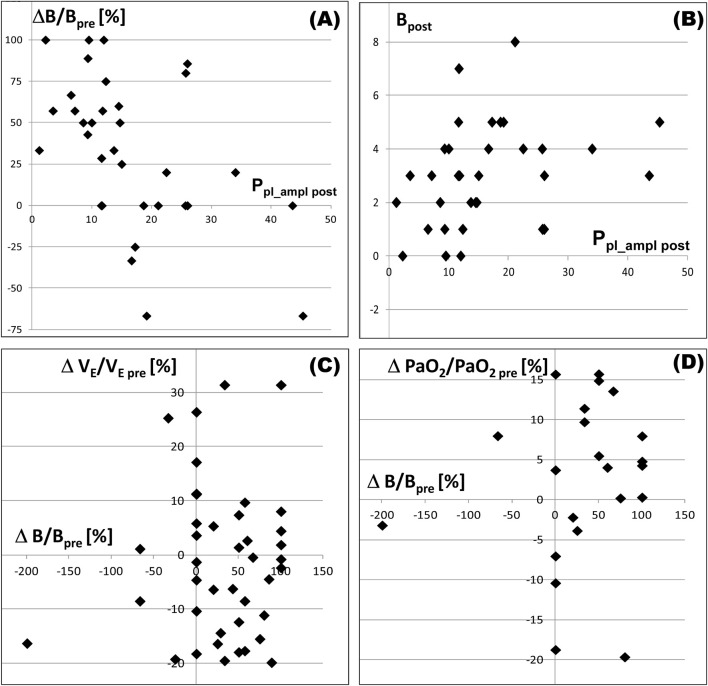
Relationships between selected parameters in patients with B_pre_>2. Subscripts “pre”and “post”indicate the value of a parameter before and after pleural fluid withdrawal, respectively; P_pl_ampl post_ – the amplitude of pleural pressure changes related to breathing (after the fluid withdrawal); **(B)** Borg score characterizing dyspnea reported by a patient; ΔB/B_pre_ – relative dyspnea relief, i.e., the decrease in B (=B_pre_-B_post_) caused by pleural fluid withdrawal expressed as percentage of B_pre_; ΔV_E_/V_E pre_ – relative minute ventilation (V_E_) increase (=V_E post_-V_E pre_) expressed as percentage of V_E pre_; ΔPaO_2_/PaO_2 pre_ – PaO_2_ increase expressed as percentage of PaO_2 pre_. **(A)** there is an inverse correlation between dyspnea relief and the amplitude; **(B)**, there is a positive correlation between dyspnea after pleural fluid withdrawal and the amplitude; **(C,D)** – dyspnea relief is associated with neither changes in V_E_ nor in PaO_2_.

P_pl_ampl post_ was the only parameter that significantly differentiated these groups: it was much greater in the NO group than in the YES group (Table 1). The association between P_pl_ampl post_ and dyspnea is additionally confirmed by the statistically significant inverse correlation between P_pl_ampl post_ and ΔB (Table 2; Figure 3A) and the significant positive correlation between P_pl_ampl post_ and B_post_ in patients reporting more considerable dyspnea before TT, i.e., in 33 patients with B_pre_>2 (Table 3; Figure 3B).

**TABLE 2:** Spearman’s correlations between changes in dyspnea (ΔB) and other parameters.

Pair of parameters	Whole sample (N = 41)	Subsample with B_pre_>2 (N = 33)
ΔB & PaO_2pre_	−0,12	−0,21
ΔB & PaO_2post_	−0,25	−0,25
ΔB & ΔPaO_2_	−0,10	−0,11
ΔB & PaCO_2 pre_	−0,06	−0,07
ΔB & PaO_2 post_	0,20	0,18
ΔB & ΔPaCO_2_	0,22	0,21
ΔB & V_E pre_	0,30	0,40^#^
ΔB & V_E post_	0,14	0,24
ΔB & ΔV_E_%	−0,17	−0,21
ΔB & P_pl_ampl post_	−0,44^#^	−0.51^#^
ΔB & V_w_	0.42^#^	0.51^#^

# statistically significant correlations. See the Table 1 caption for descriptions of the parameters.

**TABLE 3:** Other statistically significant Spearman’s correlations for the B_pre_>2 subsample.

Par. 1	Par.2	r =
B _pre_	V_E__pre_	0.38
	ΔV_E_%	−0.35 (−0.36)
	ΔPaO_2_	−0.45
P_pl_ampl post_	V_w_	−0.55 (−0.52)
	B _post_	0.36
	V_E pre_	−0.42

See the Table 1 caption for the description of the parameters. If the corresponding correlations for all patients are also statistically significant, they are shown in parentheses.

Although the minute ventilation (both V_E pre_, V_E post_ and ΔV_E_) did not differ significantly between the YES and NO groups (Table 1), V_E pre_ was statistically significantly correlated with ΔB (Table 2) and B _pre_ (Table 3) in the subsample B_pre_>2. Table 3 shows other statistically significant correlations.

## 4 Discussion

There are a number of underlying causes of dyspnea, including various cardiovascular, neuromuscular or respiratory disorders, pain, psychiatric disorders and others (Coccia et al., 2016; Beaudry et al., 2022; Ritter et al., 2024). Some of them may also be present in patients with PE. Nevertheless, since pleural fluid withdrawal usually alleviates dyspnea (the high correlation between ΔB and V_w_, Table 2), the presence of this fluid should be suspected as the main cause in patients with PE not related to congestive heart failure (dyspnea does not correlate with the pleural fluid volume in patients with cardiac-related PE (Wijayaratne et al., 2024)); however, this explains only the reason for dyspnea but does not explain its pathophysiological mechanism, i.e., the direct cause.

Changes of PaO_2_ and PaCO_2_ during TT appeared to be not associated with changes in dyspnea (Table 2); moreover, these changes were insignificant and could be both positive and negative (Table 1), even in the group YES (Figure 2B), which agrees with results of *in silico* studies (Gólczewski et al., 2025). If low PaO_2 pre_ was indeed a significant cause of considerable dyspnea before TT, more significant dyspnea relief (i.e., greater ΔB) after TT should be associated with more significant improvement of PaO_2_ (i.e., greater ΔPaO_2_) giving a significant positive correlation, which was not observed (Table 2; Figure 3D). Additionally, the higher the level of dyspnea was before TT, the less significant the PaO_2_ improvement was observed (inverse correlation between B_pre_ and ΔPaO_2_, Table 3), whereas if low PaO_2_ would be the reason for dyspnea, one could expect more significant improvement in patients with higher B_pre_. Thus, our results confirm previous suggestions that low PaO_2_ and/or elevated PaCO_2_ are not directly responsible for dyspnea in patients with PE not related to cardiovascular diseases.

In our patients with considerable dyspnea at baseline (i.e., B_pre_>2), V_E__pre_ demonstrated a statistically significant positive correlation with both B_pre_ (Table 3) and ΔB (Table 2). This might suggest that increased V_E_ could be perceived by patients as dyspnea. However, if increased V_E_ were indeed a substantial cause of dyspnea, relief of dyspnea would be associated with a decrease in V_E_. In, fact, changes in V_E_ were generally small and not associated with ΔB (Figure 3C). Furthermore, they could be both positive and negative, even in the group YES (Table 1). Additionally, ΔV_E_% exhibited a negative correlation with B_pre_ (Table 3). The above proves that more intensive ventilation could not be a substantial cause of the feeling of dyspnea in our patients.

Thus, the observed correlations (Table 2 and 3) between B _pre_ and ΔB on the one hand, and V_E pre_, ΔV_E_% and ΔPaO_2_ on the other hand were likely related to secondary associations only.

P_pl_ampl post_ was the only parameter that was significantly different between the YES and NO groups (Table 1) and was correlated with dyspnea change (ΔB in Table 2) as well as with dyspnea after TT (B_post_ in Table 3). The observed inverse correlation between ΔB and P_pl_ampl post_ (Table 2) suggests that the more intensive the work of the respiratory muscles was after TT, the smaller the relief of dyspnea was reported. Moreover, high or even very high P_pl_ampl post_ in patients in the group NO suggests that inspiratory muscles worked very effectively in these patients. This phenomenon is most likely related to the ipsilateral hemidiaphragm, as it is the main inspiratory muscle producing P_pl_ changes in this cavity (Figure 1F). Thus, on the one hand, patients from this group reported either no dyspnea relief (10 patients) or even dyspnea increase (5 patients); on the other hand, the respiratory muscles worked very effectively since they could produce such a high P_pl_ampl post_. This seems to confirm the most recent results reported by Fjaellegaard et al. (Fjaellegaard et al., 2024) that neither the normal shape nor movement of the diaphragm after TT is associated with potential dyspnea relief, as it has been suggested lately by other authors (Muruganandan et al., 2020; Skaarup et al., 2020; Muruganandan et al., 2023).

In general, WOB_e_ depends directly on P_pl_ampl_ according to the fundamental equation WOB_e_ = 0.5⋅P_pl_ampl_⋅V_E_. Thus, regardless of which respiratory muscles are responsible for such high P_pl_ampl post_ in patients in the group NO, these patients may report dyspnea due to high WOB_e_. In the YES group, WOB_e_ was significantly lower after TT, and those patients reported dyspnea relief. The association between post-TT dyspnea and post-TT WOB_e_ seems to be additionally confirmed by the correlation between B_post_ and P_pl_ampl post_ (Table 3). Note, however, that although this correlation is statistically significant, it is not very high. This may suggest that either other factors could also contribute to dyspnea or subjective feeling of increased WOB_e_ was different in individual patients or both.

Unfortunately, WOB_e_ before TT cannot be assessed on the basis of P_pl_ampl_ measured in the ipsilateral hemithorax because the ipsilateral hemidiaphragm has an insignificant contribution to respiration before TT (Figure 3B), and WOB_e_ is mostly done by the contralateral hemidiaphragm; in particular, P_pl_ampl_ in the ipsilateral hemithorax may have negative values when the ipsilateral hemidiaphragm is inverted (which would yield a ridiculous result, i.e., a ‘negative WOB_e_’). Nevertheless, WOB_e_ before pleural fluid withdrawal can be assessed on the basis of the laws of physics since, in general:

$${\text{WOB}}_{e}=0.5\cdot \text{RR}\cdot V_{T}^{2}/C_{\text{tot}}=0.5\cdot V_{E}^{2}/\left(\text{RR}\cdot C_{\text{tot}}\right)$$

where C_tot_ is the total respiratory system compliance. Thus, since neither V_E_ (Table 1) nor the median RR (Zielinska-Krawczyk et al., 2018) changes significantly during TT, changes in C_tot_ are responsible for changes in WOB_e_. Before TT, the pleural fluid causes collapse of a part of the ipsilateral lung or even the whole ipsilateral lung is not ventilated; this means that C_tot_ is reduced proportionally. According to the above formula, WOB_e_ is increased to the same extent. Moreover, lung compliance is nonlinear. Therefore, the same V_T_ ventilated smaller amount of lungs requires an additional increase of WOB_e_ (Figure 1C).

The pleural fluid compresses both the lungs and bronchi; therefore, the total WOB = WOB_e_ + WOB_r_ (where WOB_r_ is the resistive work) is additionally increased by raised work against bronchi resistance. Moreover, under physiological conditions, ribcage elastance helps inspiration, whereas additional effort is required if the thoracic cavity is expanded by PE over the volume for which the trans-wall pressure is positive at the FRC. Thus, WOB has to be increased before TT, and patients can interpret this increase as dyspnea regardless of the other possible causes.

For reasons discussed above, before TT, WOB must be increased at least owing to the collapse of a part of the lungs (Figures 1B,E), thoracic cavity expansion and narrowed bronchi. After TT, the collapsed parts can be either recruited or not. If these parts are recruited, then C_tot_ increases to the normal value (Figures 1A,D). In consequence, P_pl_ampl_ = V_T_/C_tot_ can be small, the required WOB_e_ decreases and dyspnea is reduced as in the group YES. However, if these parts remain collapsed after TT, C_tot_ is still small, and thus (Figures 1C,F).

- WOB_e_ cannot decrease and dyspnea remains,
- P_pl_ampl_ in the contralateral hemithorax has to be high because of the small C_tot_,
- as the ipsilateral hemidiaphragm is curved upward at least as much as the contralateral hemidiaphragm, high P_pl_ampl_ in the ipsilateral hemithorax, similar to P_pl_ampl_ in the contralateral hemithorax, may be generated,
- lung compliance nonlinearity may cause an increase in WOB_e_ (Figure 1C) leading to dyspnea increase, not relief.

The above can explain the lack of dyspnea relief and intriguing dyspnea increase after TT in the group NO if dyspnea in those patients before TT has been caused just by increased WOB_e_.

Concluding, either dyspnea is reduced (ΔB is large) because the WOB_e_ and P_pl_ampl post_ are small after TT (the YES group in Table 1) or WOB_e_ is still large, dyspnea persists (ΔB≤0) and P_pl_ampl post_ is high (the NO group). Certainly, some collapsed parts can be recruited, and the others remain collapsed; thus, both ΔB and P_pl_ampl_ can be moderate. As the result, a negative correlation between ΔB and P_pl_ampl post_ (Table 2) and a positive correlation between P_pl_ampl post_ and B_post_ (Table 3) are observed.

It should be noted that if the mediastinum is very compliant, P_pl_ need not be very low despite high P_pl_ampl post_ (Gólczewski et al., 2025). Indeed, in such a case, a lack of collapsed lung part recruitment requires high P_pl_ampl post_ to maintain V_E_ at a necessary level, whereas the space in the ipsilateral hemithorax that has been occupied by pleural fluid may be partly filled with the contralateral lung, which protects against excessive P_pl_ fall and seemingly suggests an expandable lung. This may partly explain the lack of differences in post-TT dyspnea relief between patients with expandable and non-expandable lung (Petersen et al., 2024). Thus, not only P_pl_ but also P_pl_ampl_ should be monitored during TT, at least to differentiate expandable and non-expandable lungs.

Although not very high, the statistically significant positive correlation between B_pre_ and V_E pre_ in the subsample B_pre_>2 (Table 3) seems to confirm that WOB_e_ is responsible for dyspnea in PE, as the greater the V_E pre_ value is, the greater the WOB_e_. It is not clear, however, why changes in dyspnea, i.e., ΔB, are not associated with ΔV_E_ (Table 2), whereas ΔV_E_ is inversely correlated with B_pre_ (Table 3). Notably, no correlations between V_E_ and arterial gas tensions were found. The relatively high inverse correlation between P_pl_ampl post_ and V_E pre_ (Table 3) has not yet been explained.

### 4.1 Study limitation

The main limitation is related to the fact that neither P_pl_ampl_ in the contralateral hemithorax nor WOB_e_ is precisely known, particularly before TT; therefore, we have had to rely on laws of physics in the interpretation of results. The fact that a study is retrospective is usually considered a limitation. In our case, however, even a prospective study would not supply better data related directly to WOB_e_ because additional measurements in patients in such poor condition would be impossible for ethical reasons. Thus, the only true limitation related to the retrospective character of this study is that TT was terminated both because of symptoms (such as too intensive cough, for example,) and when there was no more fluid or when P_pl_ excessively fell or P_pl_ampl_ increased too much (hence the inverse correlation between P_pl_ampl post_ and V_w_ in Table 3), which means some lack of sample uniformity. Certainly, the feeling of dyspnea might also be affected by other factors, such as pain, stress or fear, in patients with serious comorbidities undergoing interventional procedure; however, these factors have not been precisely recorded and, therefore, their influence cannot be analyzed here. Nevertheless, the association between WOB and dyspnea was so strong that the influence of the other causes was insufficient to destroy the correlation between P_pl_ampl post_ and dyspnea (both B_post_ and ΔB).

## 5 Conclusion

Respiratory chemoreceptors seem to control breathing effectively in patients with malignant PE since arterial blood gases do not change significantly; therefore, changes in dyspnea after pleural fluid withdrawal can be associated with neither blood gas tensions nor V_E_ changes. Our results suggest that dyspnea in PE not associated with congestive heart failure can be related to a decrease in total lung compliance (due to the collapse of a part or the whole ipsilateral lung). This decrease forces a proportional increase in WOB_e_ to maintain the ventilation and arterial gas tensions required by respiratory chemoreceptors. The strong association between dyspnea changes and P_pl_ampl post_ (statistical significance, large effect size) confirms that if pleural fluid withdrawal leads to recruitment of the collapsed parts, then both WOB_e_ and dyspnea decrease; otherwise, WOB_e_ and dyspnea do not change or can even increase owing to the nonlinearity of lung compliance. Thus, although there can be various other causes of dyspnea in an individual patient with PE, an increase in WOB should also be taken into account. Based on the analyses presented here, we recommend pleural manometry during TT, which will enable to adjust the fluid removal rate to the observed value of P_pl_ampl_.

## Data Availability

The original contributions presented in the study are included in the article/supplementary material, further inquiries can be directed to the corresponding author/s.

## Abbreviations

PE, pleural effusion; TT, therapeutic thoracentesis; Ppl, pleural pressure; AGT, arterial gas tensions; dB, ΔB, change in dyspnea, difference between the Borg Scores before and after therapeutic thoracentesis; PaO_2_, arterial oxygen partial pressure; PaCO_2_, arterial carbon dioxide partial pressure; P_pl_ampl_, amplitude of pleural pressure changes related to breathing; P_pl_ampl post_, P_pl_ampl_ after therapeutic thoracentesis; V_E_, minute ventilation; WOB, work of breathing (from the point of view of physics it is actually power, not work); WOB_e_, work of breathing which is required to overcome elastic forces; WOB_r_, work of breathing which is required to is the resistive work; RR, respiratory rate; V_T_, tidal volume; B_pre_, the Borg Scores before therapeutic thoracentesis; B_post_, the Borg Scores after therapeutic thoracentesis; V_w_, the volume of withdrawn pleural fluid; rg, the Glass rank-biserial correlation.
